# Over-Expression of LSD1 Promotes Proliferation, Migration and Invasion in Non-Small Cell Lung Cancer

**DOI:** 10.1371/journal.pone.0035065

**Published:** 2012-04-06

**Authors:** Tangfeng Lv, Dongmei Yuan, Xiaohui Miao, Yanling Lv, Ping Zhan, Xiaokun Shen, Yong Song

**Affiliations:** 1 Department of Respiratory Medicine, Jinling Hospital, Nanjing University School of Medicine, Nanjing, China; 2 First Department of Respiratory Medicine, Nanjing Chest Hospital, Nanjing, China; National Taiwan University Hospital, Taiwan

## Abstract

**Background:**

Lysine specific demethylase 1 (LSD1) has been identified and biochemically characterized in epigenetics, but the pathological roles of its dysfunction in lung cancer remain to be elucidated. The aim of this study was to evaluate the prognostic significance of LSD1 expression in patients with non-small cell lung cancer (NSCLC) and to define its exact role in lung cancer proliferation, migration and invasion.

**Methods:**

The protein levels of LSD1 in surgically resected samples from NSCLC patients were detected by immunohistochemistry or Western blotting. The mRNA levels of LSD1 were detected by qRT-PCR. The correlation of LSD1 expression with clinical characteristics and prognosis was determined by statistical analysis. Cell proliferation rate was assessed by MTS assay and immunofluorescence. Cell migration and invasion were detected by scratch test, matrigel assay and transwell invasion assay.

**Results:**

LSD1 expression was higher in lung cancer tissue more than in normal lung tissue. Our results showed that over-expression of LSD1 protein were associated with shorter overall survival of NSCLC patients. LSD1 was localized mainly to the cancer cell nucleus. Interruption of LSD1 using siRNA or a chemical inhibitor, pargyline, suppressed proliferation, migration and invasion of A549, H460 and 293T cells. Meanwhile, over-expression of LSD1 enhanced cell growth. Finally, LSD1 was shown to regulate epithelial-to-mesenchymal transition in lung cancer cells.

**Conclusions:**

Over-expression of LSD1 was associated with poor prognosis in NSCLC, and promoted tumor cell proliferation, migration and invasion. These results suggest that LSD1 is a tumor-promoting factor with promising therapeutic potential for NSCLC.

## Introduction

Lung cancer is one of the leading causes of cancer death worldwide. Non-small cell lung cancer (NSCLC) is the most common type of lung cancer [1]. The 5-year survival rate for lung cancer remains poor. In order to develop more effective therapies, it is important to obtain a better understanding of the molecular biology of lung cancer.

Genetic alterations are a hallmark of human cancer. In recent years, the cancer genomics field has made significant advances in identifying genetic lesions in cancer. Furthermore, the importance of epigenetic changes that occur during lung cancer development has also been recognized [2]. Epigenetic changes are associated with both DNA methylation and histone modifications [3]. Histone modifications, such as acetylation, phosphorylation and methylation, are the switches that alter chromatin structure to allow posttranscriptional activation or repression of downstream proteins [4]. Understanding these epigenetic changes will identify novel cancer-related genes that may represent attractive targets for cancer treatment and provide new insights into the biology of lung cancers. Thus, an integrative approach in lung cancer research, combining epidemiological, genetic and epigenetic information, has emerged as an important concept for cancer therapy [5].

The methylation status of histone methyltransferases and histone demethylases plays a pivotal role in the regulation of gene expression [6]. Histone demethylase lysine specific demethylase 1 (LSD1), the first histone demethylase that was discovered as a nuclear homolog of amine oxidases, removes the methyl groups from mono- and dimethylated Lysine (Lys)4 of histone H3 (H3K4me1/2) and Lys9 of histone H3 (H3K9me1/2) [7]. LSD1 is essential for mammalian development and involved in many biological processes, such as cell-type differentiation and gene activation and repression [8]. A recent study indicated that LSD1 might promote cell phase transition (deficiency in LSD1 led to partial cell cycle arrest in G_2_/M) and cell proliferation, suggesting that its over-expression might promote tumorigenesis [9]. The expression of LSD1 has been associated with tumor recurrence during therapy in various cancers, further implicating LSD-1 as a tumor promoter [10]–[12]. Tissue cDNA microarray analysis also revealed LSD1 transactivation in lung and colorectal carcinomas [11]. Knocking down of LSD1 with small interfering (si)RNAs resulted in suppression of proliferation of various bladder and lung cancer cell lines [11]. However, although these studies demonstrated that LSD1 may be associated with the pathogenesis of lung cancer, the expression and significance of LSD1 in NSCLC is obscure.

In this study, we attempted to investigate the expression and function of LSD1 in NSCLC, its relationship with clinicopathological features, and its prognostic value for survival of patients with NSCLC. Finally, we also aimed to determine the exact role of LSD1 in lung cancer proliferation, migration and invasion.

## Materials and Methods

### Patients and Specimens

Surgical specimens from 80 NSCLC patients obtained at the Nanjing Chest Hospital and the Jinling Hospital from January 2001 to December 2003 were retrospectively collected for study. These consisted of 38 squamous carcinomas and 42 adenocarcinomas, along with patient-matched adjacent non-tumor tissue specimens. None of the patients had received radiotherapy or chemotherapy prior to surgery. All patients had been followed-up for five years after operation, and complete clinical data were electronically recorded. All tumor tissues were classified according to the World Health Organization classification guidelines. The staging of the tumors followed the 7^th^ Union International Classification at Cancer criteria defined in 2009. This study was approved by the Ethical Committee of the Jinling Hospital at Nanjing. All samples were anonymized, and none of the researchers conducting the experiments had access to the clinicopathological data.

### Immunohistochemical (IHC) Staining

All samples were fixed in 10% formalin, embedded in paraffin, sectioned consecutively at 5 µm, and stained by hematoxylin and eosin. The sections were deparaffinized and rehydrated according to routine protocol. The sections were incubated for overnight with the specific primary LSD1 antibodies (1∶100 dilution; Cell Signaling, Danvers, MA, USA). The slides were incubated for 30 min with goat anti-rabbit immunoglobulins (E0432; Cell Signaling) after being washed with Tris-buffered NaCl solution. The slides were then incubated for 30 min with stripped AB complex/AP (K0391; Dako, Peking, China). Counterstaining was performed with hematoxylin. The percentage of positive cells was determined by counting 500 cells in five random areas per section. Nuclear immunostaining results for LSD1 were evaluated using a semi-quantitative Remmele scoring system [13], which calculated the staining intensity and the percentage of positive cells. IHC staining was scored according to the following criteria: –, none of the cells stained; +, 1–40% of the cells stained; ++; 40–70% of the cells stained; +++, 70–100% of the cells stained. IHC score of LSD1 expression was [0 (negative) ≤ score < 2+] and [2+ ≤ score ≤3+], which represented low and high expression, respectively.

### Western Blotting

The lung cancer tumor tissues and adjacent non-tumor tissues were homogenized. Total proteins (30 µg) from clinical specimens or cell cultures were separated by sodium dodecyl sulfate-polyacrylamide gel electrophoresis, followed by electrotransfer to a nitrocellulose membrane by use of a transfer cell (Bio-Rad, Hercules, CA, USA). Western blotting was carried out by sequential incubation in 5% non–fat milk blocking buffer at room temperature for 60 min, followed by primary antibody against either LSD1 (1∶500; Cell Signaling), AcH3K9, Twist1, E-cadherin, N-cadherin or β-actin at 4°C overnight, and finally horseradish peroxidase-conjugated anti-rabbit secondary antibody (1∶5000) at room temperature for 90 min. Immunoreactive bands were detected by reaction with the ECL detection system reagents (Amersham, Arlington Heights, IL, USA) and exposure to X-ray film, which was the developed and photographed.

### mRNA Extraction and Quantitative Reverse Transcription (qRT)-PCR

Tissues were homogenized and total cellular mRNA was isolated using the TRIzol reagent (Invitrogen, Carlsbad, CA, USA). The cDNA was transcribed using M-MLV reverse transcriptase (Promega, Madison, WI, USA), according to the manufacturer’s protocol. qRT-PCR was performed using the FastStart Universal SYBR Green Master Mix kit (Roche, San Francisco, CA, USA). Relative mRNA levels of LSD1 were normalized to levels of the housekeeping gene GAPDH and calculated by the 2-ΔΔCt method. The following primers were used: GAPDH, forward: 5'-CCATGTTCGTCATGGGTGTGAACCA-3' and reverse: 5'-GCCAGTAGAGGCAGGGATGATGTTG-3'; LSD1, forward: 5'-GAAACTGGAATAGCAGAGAC-3' and reverse: 5'-GGTGGACAAAGCACAGTATCA-3'.

### Cell Culture, Transfection and Treatment

A549, H460, and 293T cells were obtained from the American Type Culture Collection (Manassas, VA, USA) and cultured in RPMI medium supplemented with 10% fetal bovine serum (FBS), 2 mM L-glutamine and 100 µg/mL penicillin/streptomycin. The plasmid Flag-LSD1 (3ug); kindly provided by Dr. Yang Shi, University of Southern California, Los Angeles, CA, USA), over-expression plasmid LSD1 (3 µg) and LSD1 siRNA (3 µg) were transfected into A549 and H460 cell lines in 6-well plates (1 µg/mL) using the Lipofectamine reagent (Invitrogen), according to the manufacturer’s instructions. Pargyline (Sigma-Aldrich, St. Louis, MO, USA), a monoamine oxidase inhibitor, was used to chemically block LSD1-mediated demethylation [14]. Cells were treated with pargyline for 48 h, and then were subjected to MTS assay. The data are presented as fold-changes in relative light units/milliunits of pargyline, and represent the average of three independent experiments.

### Cell Proliferation, Growth, MTS Assays

For cell growth assay, 50,000 cells/well were seeded in triplicate in a 24-well plate with complete growth medium. Cells were counted over six days using a hemocytometer. For the MTS assay (Promega), 500 cells/well were seeded in triplicate in a 96-well plate for each cell line. At 12, 24, and 48 h, 20 µL of MTS reagent was added to each well, incubated for 1 h at 37°C, and the results were analyzed by a plate reader at 490 nm. The sample data was normalized to background readings of media only.

### Three-dimensional Cell Culture, Collagen Invasion Assay, and Scratch Assay

Three-dimensional basement membrane cultures were established as previously described [15]. Briefly, 5000 cells/well were grown in 2% matrigel (BD Biosciences, San Jose, CA, USA) with epidermal growth factor (EGF) on a 50% matrigel base layer. The collagen invasion assay was performed as previously described [16]. Briefly, 5000 cells/well were seeded in 2% matrigel on a layer of 1∶1 collagen (BD Bioscience) to matrigel mix. Culture growth was recorded on day 5. For the scratch assay, cells were grown in complete growth medium until 90–100% confluency was reached. A 3 mm wound was introduced across the diameter of each plate. Cell migration was observed by microscopy at 24 and 48 h later.

**Figure 1:**
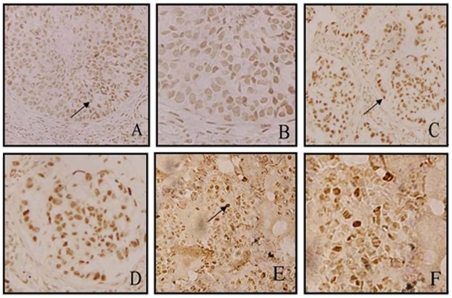
LSD1 expression and distribution in NSCLC tumor tissues and normal lung tissues, detected by IHC staining. ( A, B) LSD1 expression was up-regulated in lung squamous cancer tissue. The arrows indicate LSD1-positive cancer cells, which were located in the cell nuclei. Magnification: A, ×20; B, ×60. (C, D) LSD1 expression was up-regulated in lung adenocarcinoma cancer tissue. Magnification: C, ×20; D, ×60. (E, F) IHC revealed that LSD1 expression was weak in normal lung tissues. Magnification: E, ×20; F, ×60. IHC scores of LSD1 expression were: low, [0 (negative) ≤ score < 2+]; high, [2+ ≤ score ≤3+]. *P*<0.05 indicated statistically significant differences between groups.

**Figure 2:**
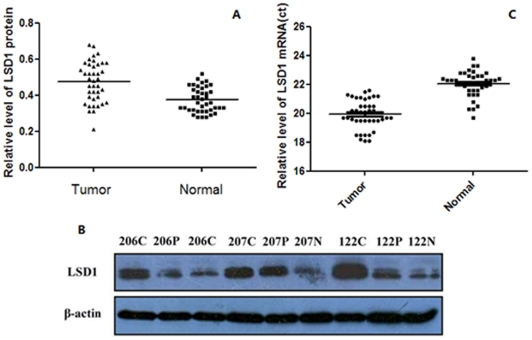
Up-regulation of LSD1 expression in NSCLC tumor tissues. (A) The protein level of LSD1 in lung cancer tissue was significantly higher than in normal tissue (*P*<0.01), as detected by Western blot. β-tubulin was used as a loading control. (B) Sample 122, 206 and 207 are shown as representatives of the three groups: N, normal lung tissues: C, NSCLC tissues; P, paracarcinoma tissues. (C) The mRNA level of LSD1 was detected by qRT-PCR, and the mRNA expression of LSD1 was significantly higher in tumor tissue than in normal tissue (*P*<0.05).

**Table 1:** Statistical analysis of LSD1 expression levels in clinical lung tissues.

LSD1						
Factor	Cases, n	Negative	Low	High	κ^2^	*P*
**Age, years**	80	10	43	27	0.007	0.934
<60	38	3	22	13		
≥60	42	7	21	14		
**Gender**					0.553	0.460
Male	61	10	31	20		
Female	19	0	11	8		
**Smoker**					0.760	0.180
No	41	2	21	17		
Yes	39	8	20	11		
**Histology**					0.007	0.934
Squamous carcinoma	38	6	19	13		
Adenocarcinoma	42	7	21	14		
**Pathological stage**					0.030	0.870
T<3cm	18	1	11	6		
T≥3cm	62	9	31	22		
**Nodal status**					0.005	0.940
N0	49	6	26	17		
N1-2	31	4	16	11		
**OS, months**					12.870	0.0003
≤12	14	2	6	6		
12–36	27	3	8	16		
36–60	11	2	5	4		
>60	28	2	24	2		

**Figure 3:**
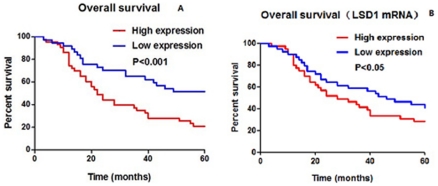
LSD1 expression was associated with overall survival in NSCLC. Blue lines depict high levels of LSD1 expression, and red lines depict low levels of LSD1 expression in NSCLC samples. (A) NSCLC patients with lower LSD1 protein levels had better prognosis. (unadjusted *P*-value = 0.0003; low expression cases, n = 52 and high expression cases, n = 28). (B) Patients with lower mRNA levels of LSD1 also had longer survival (*P*<0.05).

### Statistical Analysis

Student’s *t*-test and ANOVA were used to investigate the associations between LSD1 expression and clinical factors (age, sex, smoking, tumor stage, histology, tumor size, nodal status, and overall survival). Pearson’s correlation analysis was used to evaluate the degree of linear correlation between two variables. Kaplan-Meier survival analysis was performed to determine the prognostic value of LSD1, and log-rank test was used to compare the equality of the two survival curves. A *P*-value <0.05 was considered statistically significant and *P*<0.01 was considered highly significant. All statistical analyses were performed using the SPSS program v17.0 for Windows (SPSS, Chicago, IL, USA).

## Results

### LSD1 Expression was Up-regulated in NSCLC Tumor Tissues

We first examined expression levels of LSD1 in 80 NSCLC tumor tissues and 20 normal lung tissues by IHC staining. High LSD1 expression was detected in the nuclei of malignant cells, while weak staining was observed throughout the non-neoplastic tissues (Fig. 1). Specifically, the expression of LSD1 was observed in 90.0% of lung carcinomas (72/80) and 10.0% of benign lung specimens (2/20). High levels of LSD1 expression (score: ++-+++) were detected in 37 (46.3%) tumor tissues from NSCLC patients.

We next examined LSD1 expression in 40 randomly selected NSCLC tumor tissues, paracarcinoma lung tissues, and normal lung tissues by Western blotting. The results revealed a statistically significant elevation of LSD1 expression in tumors, as compared to the normal lung tissues using β-tubulin as the reference (two-tailed paired *t*-test, n = 40, *P*<0.01) (Fig. 2A). The protein level of LSD1 in samples 122, 206, and 207 is shown in Figure 2B.

To further validate the above protein results, we also examined the mRNA levels of LSD1 in these 40 selected NSCLC tumor tissues and normal lung tissues by qRT-PCR using GAPDH as reference. We found that the mRNA level of LSD1 in cancer tissues (Ct = 19.9±1.01) was significantly higher than that in normal lung tissues (Ct = 22.1±0.9) (two-tailed paired *t*-test, n = 40, *P*<0.05) (Fig. 2C).

### LSD1 Expression was Associated with Overall Survival

To evaluate the clinical significance of LSD1 over-expression in lung cancer, we analyzed whether expression levels of LSD1 were associated with overall survival in NSCLC. As shown Table 1, high protein levels of LSD1 were significantly associated with survival (χ^2^ = 12.87, *P* = 0.0003), but not correlated with any of the clinicopathologic characteristics (including age, smoking habits, sex, tumor diameter, histologic type, visceral pleural invasion, pathologic stage, or type of operation; all, *P*>0.05). During the 5-year follow-up period, 52 out of 80 (65%) NSCLC patients died as a result of disease progression. Kaplan–Meier curves indicated that patients with increased LSD1 expression (28 cases) had a significantly shorter overall survival than those with decreased LSD1 expression (52 cases) (*P* = 0.0003) (Fig. 3A).

**Figure 4:**
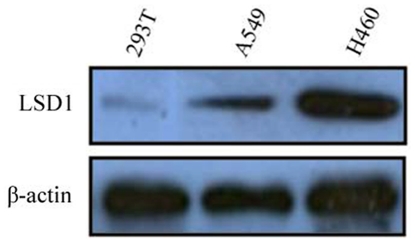
Expression of LSD1 in A549, H460 and 293T cells. The expression of LSD1 in the A549 and H460 lung cancer cells was higher than in the 293T cells. The protein level in the H460 cell line was higher than that in the A549 cell line. β-actin was used as loading control.

**Figure 5:**
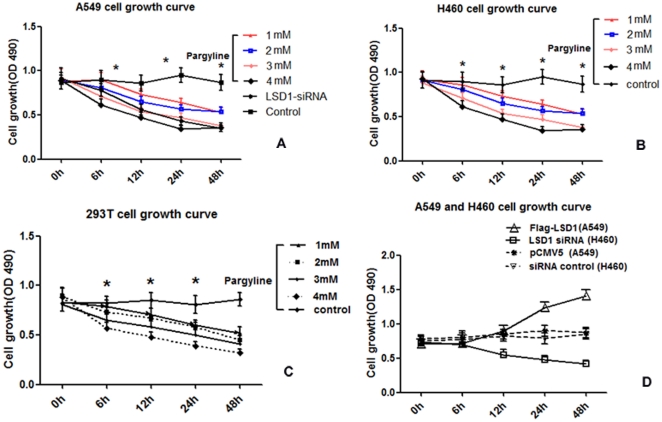
The proliferation curve of A549, H460 and 293T cell lines after pargyline treatment and transfection of Flag-LSD1 or LSD1 siRNA. The series of curves represent cell proliferation at 0 h to 48 h after exposure to different concentrations of pargyline treatment and transfection with the different plasmids. (A) The LSD1 inhibitor, pargyline (from top to bottom: 1mM, 2mM, 3mM and 4mM), and LSD1 siRNA inhibited proliferation of the A549 cell line. The A549 cell proliferation was significantly decreased in the different groups, as compared to the controls (*P<0.05). (B) Pargyline inhibited H460 cell line survival. Moreover, a stronger inhibitory effect was observed in the H460 cell line, but there were no significant differences between the two cell lines. The H460 cell proliferation was significantly decreased in the different concentration groups, as compared to the controls (*P<0.05). (C) Pargyline inhibited the survival of the 293T cell line. (D) The proliferation curve after transfection of Flag-LSD1 plasmid into the A549 cell and transfection of LSD1 siRNA plasmid into the H460 cell. In A549 cells, the proliferation was significantly higher after transfection of the Flag-LSD1 plasmid, as compared to the pCMV5-transfected control (*P<0.05). In H460 cells, the proliferation was significantly lower after transfection of LSD1 the siRNA plasmid, as compared to the cells transfected with siRNA control plasmid (*P<0.05).

**Figure 6:**
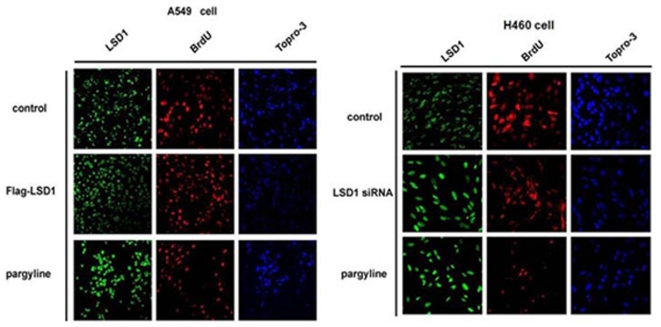
Changes in LSD1 activity affect cell proliferation in the A549 and H460 cell lines, as detected by immunofluorescence. Images are shown at ×20 magnification. Green, red and blue fluorescence represent LSD1, BrdU and Topro-3, respectively. The proliferation significantly increased in the A549 cells after transfection of the Flag-LSD1 plasmid. However, proliferation decreased in the H460 cells after transfection of the LSD1 siRNA plasmid. Pargyline treatment reduced proliferation in both cell lines. Topro-3 staining shows nuclear localization, and BrdU staining shows the proliferating cells.

**Figure 7:**
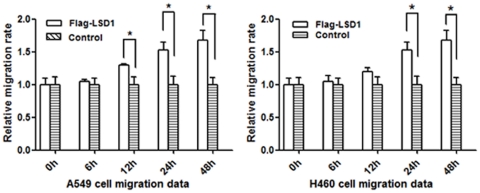
The relative velocity change of cell migration in A549 and H460 cells transfected with Flag-LSD1 or LSD1 siRNA plasmid, respectively. The migration velocity gradually decreased from 6 h to 48 h after transfection of the LSD1 siRNA plasmid in the H460 cells, which was significantly different from the velocity at 24 h and 48 h (*P<0.05). The migration rate gradually increased after transfection of Flag-LSD1 plasmid into the A549 cells, which was significantly different from the velocity at 24 h and 48 h (*P<0.05). All transfection groups were significant different from the control groups (*P<0.05).

In agreement with this, we also determined that higher mRNA expression of LSD1 was associated with decreased overall survival in NSCLC patients (*P*<0.05) (Fig. 3B.).

### LSD1 Promoted Proliferation in Lung Cancer Cell Lines

The expression of LSD1 was lower in the A549 cell than in the H460 cell line, as shown in Figure 4. Therefore, the Flag-LSD1 plasmid was transfected into the A549 cells and the LSD1 siRNA plasmid was transfected into the H460 cells. The 293T normal epithelial cells were used as a negative control.

We detected the OD value of A549, H460 and 293T cells by MTS to generate cell growth curves. In all three cell lines, the cellular proliferation declined along with pargyline treatment in a concentration-dependent manner. It was notably lower in the 4 mM-treated group than in the 1 mM- or 2 mM-treated groups. In addition, it was also lower in the 1 mM-treated group than in the 3 mM-treated group; however, there was no difference between the 1 mM- and 2 mM-treated groups or the 3 mM- and 4 mM-treated groups (*P*>0.05), as shown in Figure 5A-C.

**Figure 8:**
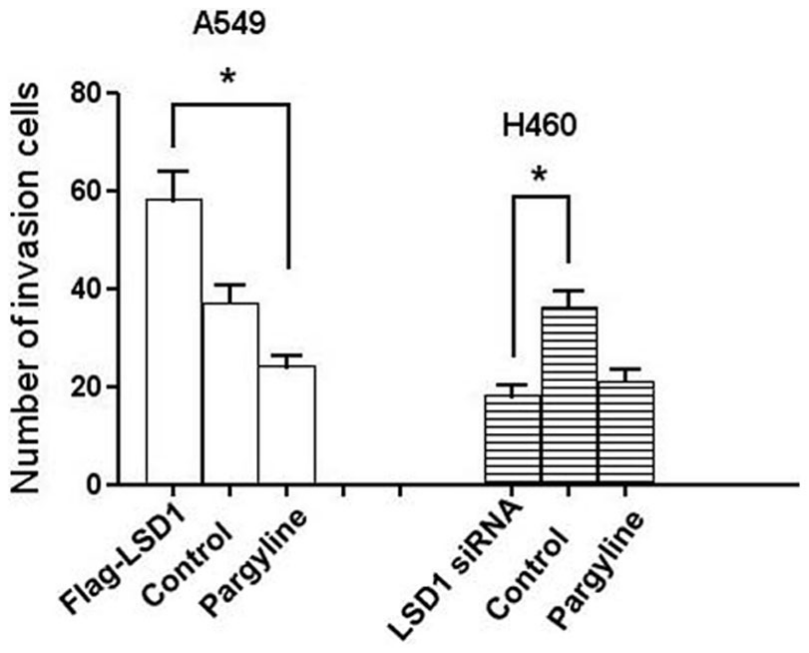
The numbers of A549 and H460 cells that transversed the matrigel basement membrane. The transversed cell amount was significantly higher for A549 cells transfected with the Flag-LSD1 plasmid; however, transfection of LSD1 siRNA plasmid into the H460 cells led to decreased amounts of invasive cells. The numbers of invasive cells from both transfection groups were significant different from the control group (* *P*<0.05).

**Figure 9:**
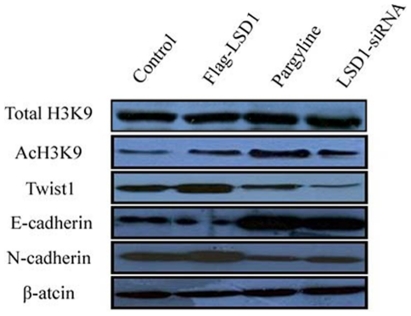
Changes in LSD1 activity affect AcH3K9, Twist1, E-cadherin and N-cadherin expression in A549 and H460 cells. The expression of total H3K9 did not change remarkably. When LSD1 activity was up-regulated, Twist1 and N-cadherin expression increased and AcH3K9 and E-cadherin expression decreased. When LSD1 activity was down-regulated, Twist1 and N-cadherin expression decreased and AcH3K9 and E-cadherin expression increased. The Flag-LSD1 plasmid was transfected into the A549 cell line, and the LSD1 siRNA plasmid was transfected into the H460 cell line. β-actin was used as loading control.

The proliferation significantly increased in the A549 group after transfection of the Flag-LSD1 plasmid (*P*<0.05); however, it significantly decreased in the H460 group after transfection of the LSD1 siRNA plasmid (*P*<0.05). The pCMV plasmid was used as the control in the A549 transfection group, and the LSD1 siRNA control plasmid was used in the H460 cell transfection group (Fig. 5D).

We further identified the role of LSD1 in A549 and H460 cell proliferation by immunofluorescence staining. The change in number of proliferating cells was observed by BrdU staining. The results demonstrated that the cell proliferation was significantly increased after transfection of the over-expressing LSD1 plasmid into the A549 cell line. Meanwhile, cell proliferation decreased in the H460 cells transfected with the LSD1 siRNA plasmid. It decreased in both cell lines after pargyline treatment, as shown by immunofluorescence staining (Fig. 6), which also confirmed that LSD1 is involved in tumor cell proliferation.

### LSD1 Promoted the Migration of Lung Cancer Cells

Cell migration was investigated by the cell scratch assay. Compared with the control group, the number and rate of migrated cells was gradually reduced after transfection with LSD1 siRNA plasmid in the H460 cell lines. The migrating cells reached a peak at 48 h, as shown in Figure 7A. In contrast, the number of migrated cells were significantly increased in the A549 cell line after transfection with Flag-LSD1 plasmid, as compared to the control group (*P*<0.05). The relative speed of migration is shown in Figure 7B.

### The Role of LSD1 in Lung Cancer Invasion

The ability of cells to cross matrigel indicated the invasiveness of the A549 and H460 cell lines. H460 cells transfected with LSD1 siRNA plasmid were less invasive than the control group (*P*<0.05). A549 cells transfected with Flag-LSD1 plasmid were more invasive than the control cells and cells treated with pargyline (both, *P*<0.05). The amount of invading cells crossing through the matrigel basement membrane is shown in Figure 8.

### LSD1 Regulated Epithelial-to-mesenchymal Transition (EMT) in Lung Cancer Cells

To investigate whether LSD1 regulates the EMT transition in lung cancer cells, we examined the expression of the key EMT transcriptional repressor TWIST1 and the EMT markers E-cadherin and N-cadherin in LSD1-over-expressing A549 cells (transfected with the Flag-LSD1 LSD1 over-expressing plasmid) and LSD1 knocked down H460 cells (transfected with the LSD1 siRNA plasmid, or treated with 4 mM pargyline). The effects on H3K9, AcH3K9, Twist1, E-cadherin and N-cadherin expression induced by alteration of the LSD1 activity were examined. While expression of total H3K9 was unaffected, LSD1 over-expression led to decreases in H3K9 acetylation and E-cadherin expression and increases in Twist1 and N-cadherin expression. Meanwhile, H3K9 acetylation levels and E-cadherin expression were increased in LSD1 knock down cells, suggesting that LSD1 expression might be correlated with AcH3K9, Twist1, E-cadherin and N-cadherin expression, as shown in Figure 9.

## Discussion

In recent years, epigenetics has become a hot topic in cancer research. The balance of methylation and demethylation in epigenetic modification affects gene expression and cellular activity. Studies have demonstrated that aberrant histone lysine methylation in cancer is associated not only with the repression of chromatin related to specific genes, but also with the repression of large chromosomal regions. Epigenetic changes in LSD1 have been shown to play a key role in carcinogenesis [17]. LSD1 can prevent the accumulation of the dimethyl groups of p53, repressing p53-mediated transcriptional up-regulation, preventing apoptosis, and contributing to human carcinogenesis via a chromatin modification mechanism.

To date, only a few studies have implicated LSD1 in NSCLC. Hayami *et al.* demonstrated that LSD1-specific siRNAs significantly knocked down LSD1 expression and resulted in suppressed proliferation of various lung cancer cells [11]. However, the association between LSD1 and the survival of NSCLC patients was not well defined, and the role of LSD1 in proliferation, migration and invasion in NSCLC was obscure. Our study investigated the associations of LSD1 expression and clinical features of NSCLC patients diagnosed as stage I/II. In order to demonstrate that the epigenetic changes were associated with genetic changes in lung cancer, we first investigated the expression of LSD1 in NSCLC clinical samples.

Previous studies demonstrated that LSD1 protein and mRNA levels could act as biomarkers for patients with more aggressive tumors of breast cancer, prostate cancer, and neuroblastoma [18]–[20]. In our study, we detected LSD1 by IHC analysis, Western blotting, and qRT-PCR. A strong point of this research is the finding that expression of LSD1 was significantly correlated with overall survival of NSCLC patients. Our current research investigated the role of LSD1 in NSCLC. Further studies on LSD1 in other tumor types may reveal that patients with higher levels of LSD1 (regardless of mRNA or protein and/or regardless of primary tumor type) have poorer survival than patients with lower levels of LSD1.

We further demonstrated that LSD1 was important for cancer cell proliferation and invasion. LSD1-induced activation of proliferation, migration and invasion in tumor cell was inhibited by pargyline. Down-regulation of LSD1 expression by siRNA in tumor cell lines led to increased cell growth, migration and invasion. These data suggest that LSD1 may influence the transformation of tumor cells and may also promote EMT in the lung epithelium. Further research needs to be conducted in order to determine whether over-expression of LSD1 is an early or late event in lung tumorigenesis and what the mechanism of over-expression is. We have presented data suggesting that LSD1 is up-regulated in the lung cancer tumor promotion pathway and that it acts to increase cell growth, migration and invasion, and to restore a transformed epithelial phenotype. Pharmacological suppression of LSD1 may represent a promising approach for NSCLC treatment.

Further functional analysis is required to determine the regulatory network of LSD1. Transcriptional analysis revealed that the LSD1/NuRD complexes regulate several cellular signaling pathways [21], including the TGFβ1 signaling pathway that is critically involved in cell proliferation, survival, and epithelial-to-mesenchymal transition. We demonstrated that LSD1 promoted the invasion and metastatic potential of lung cancer cells *in vitro*. We also found that LSD1 is up-regulated in lung carcinomas and that its level of expression is negatively correlated with that of AcH3K9, Twist1 and N-cadherin, and positively correlated with that of E-cadherin. Our data provide a molecular basis for the interplay of histone deacetylation in chromatin remodeling, indicating that LSD1 may affect the EMT and acetylation of H3K9.

The aberrant over-expression of LSD1 in lung cancer may make it a good candidate as a therapeutic molecular target [11]. This kind of information also indicates that we should carry on the development of novel anticancer therapy based on epigenetic status. As it has implications for the discovery of epigenetic markers, an important question to resolve is how to define potential targets for epigenetic therapy. First-generation drugs targeting the relatively promiscuous DNA methylation and histone acetylation modifiers have had success in the treatment of hematological cancers [22]. If LSD1 inhibition leads to significant de-repression of some genes, LSD1 might be an important alternative target for therapy. Epigenetic control of gene regulation is a rapidly developing field with substantial potential, and oncology is likely to be the therapeutic application in which the fastest progress is made. To date, synthetic inhibitors of classical histone deacetylases have been widely used as biological tools for epigenetic studies, and some have advanced to clinical studies. In addition, development of histone methyltransferase and demethylase inhibitors has recently been reported [21], [23]. In lung cancer, over-expression of LSD1 should contribute to gene repression to inhibit cellular growth and malignant progression, but where there is an actual purpose of LSD1 remains unknown. We plan to investigate this in future studies.

In conclusion, we found that LSD1 was over-expressed in NSCLC patients, through early to late stages of carcinogenesis. LSD1 is present in the nucleus and promotes proliferation, possibly through regulation of a wide variety of chromatin functions. Meanwhile, over-expression of LSD1 promoted tumor cell proliferation, migration and invasion. Further validation with functional analyses of this protein in the context of human carcinogenesis may assist in development of novel therapeutic strategies for lung cancer.

## References

[1] Jemal A, Siegel R, Xu J, Ward E (2010). Cancer statistics, 2010.. CA Cancer J Clin.

[2] Piperi C, Vlastos F, Farmaki, Martinet N, Papavassiliou AG (2008). Epigenetic effects of lung cancer predisposing factors impact on clinical diagnosis and prognosis.. J Cell Mol Med.

[3] Seligson DB, Horvath S, McBrian MA, Mah V, Yu H (2009). Global levels of histone modifications predict prognosis in different cancers.. Am J Pathol.

[4] Yang S (2007). Histone lysine demethylases: emerging roles in development, physiology and disease.. Nature Reviews, Genetics.

[5] Risch A, Plass C (2008). Lung cancer epigenetics and genetics.. Int J Cancer.

[6] Ueda R, Suzuki T, Mino K, Tsumoto H, Nakaqawa H (2009). Identification of Cell-Active Lysine Specific Demethylase 1-Selective Inhibitors.. J Am Chem Soc.

[7] Shi Y, Lan F, Matson C, Mulliqan P, Whetstine JR (2004). Histone demethylation mediated by the nuclear amine oxidase homolog LSD1.. Cell.

[8] Wang J, Scully K, Zhu X, Cai L, Zhang J (2007). Opposing LSD1 complexes function in developmental gene activation and repression programmes.. Nature.

[9] Scoumanne A, Chen X (2007). The lysine-specific demethylase 1 is required for cell proliferation in both p53-dependent and -independent manners.. J Biol Chem.

[10] Lim S, Janzer A, Becker A (2010). Lysine-specific demethylase 1 (LSD1) is highly expressed in ER-negative breast cancers and a biomarker predicting aggressive biology.. Carcinogenesis.

[11] Hayami S, Kelly JD, Cho HS, Yoshimatsu M, Unoki M (2011). Overexpression of LSD1 contributes to human carcinogenesis through chromatin regulation in various cancers.. Int J Cancer.

[12] Chen PP, Li WJ, Wang Y, Zhao S, Li DY (2007). Expression of Cyr61, CTGF, and WISP-1 correlates with clinical features of lung cancer.. PLoS One.

[13] Remmele W, Stegner HE (1987). Recommendation for uniform definition of an immunoreactive score (IRS) for immunohistochemical estrogen receptor detection (ER-ICA) in breast cancer tissue.. Pathologe.

[14] Metzger E, Wissmann M, Yin N, Müller JM, Schneider R (2005). LSD1 demethylates repressive histone marks to promote androgen-receptor-dependent transcription.. Nature.

[15] Debnath J, Muthuswamy SK, Brugge JS (2003). Morphogenesis and oncogenesis of MCF-10A mammary epithelial acini grown in three-dimensional basement membrane cultures.. Methods.

[16] Xiang B, Muthuswamy SK (2006). Using three-dimensional acinar structures for molecular and cell biological assays.. Methods Enzymol.

[17] Lim S, Metzger E, Schüle R, Kirfel J, Buettner R (2010). Epigenetic regulation of cancer growth by histone demethylases.. Int J Cancer.

[18] Lim S, Janzer A, Becker A, Zimmer A, Schüle R (2010). Lysine-specific demethylase 1 (LSD1) is highly expressed in ER-negative breast cancers and a biomarker predicting aggressive biology.. Carcinogenesis.

[19] Schulte JH, Lim S, Schramm A, Friedrichs N, Koster J (2009). Lysine-specific demethylase 1 is strongly expressed in poorly differentiated neuroblastoma: implications for therapy.. Cancer Res.

[20] Suikki HE, Kujala PM, Tammela TL, van Weerden WM, Vessella RL (2010). Genetic alterations and changes in expression of histone demethylases in prostate cancer.. Prostate.

[21] Wang Y, Zhang H, Chen Y, Sun Y, Yang F (2009). LSD1 is a subunit of the NuRD complex and targets the metastasis programs in breast cancer.. Cell.

[22] Best JD, Carey N (2010). Epigenetic opportunities and challenges in cancer.. Drug Discov Today.

[23] Cole PA (2008). Chemical probes for histone-modifying enzymes.. Nat Chem Biol.

