# Expression of Concern: Over-Expression of LSD1 Promotes Proliferation, Migration and Invasion in Non-Small Cell Lung Cancer

**DOI:** 10.1371/journal.pone.0298436

**Published:** 2024-02-02

**Authors:** The PLOS ONE Editors

After this article [1] was published, the following issues were noted:

- In Fig. 4, when levels are adjusted to visualize background, there appears to be a vertical discontinuity in the LSD1 panel between the 293T and A549 lanes.
- The results shown in Fig. 7 do not correspond to the description in the Figure legend or the corresponding text of the Results section. Specifically, the figure does not report data for an LSD1 siRNA experiment and so the following result statement is not supported: “*Compared with the control group*, *the number and rate of migrated cells was gradually reduced after transfection with LSD1 siRNA plasmid in the H460 cell lines*.”
- The article does not report sufficient detail about the controls used in the Flag-LSD1 and LSD1 siRNA transfection experiments to replicate these experiments.
- Similarities were noted between this article [1] and work by other research groups which call into question the validity and provenance of the reported results.

The authors did not respond or could not be reached to discuss these issues.

The *PLOS ONE* Editors issue this Expression of Concern to inform readers of the unresolved issues.
